# Socio-Economic and Health Literacy Inequalities as Determinants of Women’s Knowledge about Their Reproductive System: A Cross-Sectional Study

**DOI:** 10.3390/epidemiologia5040044

**Published:** 2024-09-26

**Authors:** Viktória Prémusz, Kálmán András Kovács, Eszter Skriba, Zoltán Tándor, Gábor Szmatona, Olívia Dózsa-Juhász

**Affiliations:** 1 Faculty of Health Sciences, University of Pécs, 7621 Pécs, Hungary; olivia.juhasz@etk.pte.hu; 2 MTA-PTE Human Reproduction Scientific Research Group, University of Pécs, 7624 Pécs, Hungary; kovacs.kalman@pte.hu; 3 National Laboratory on Human Reproduction, University of Pécs, 7622 Pécs, Hungary; 4 Doctoral School of Health Sciences, University of Pécs, 7621 Pécs, Hungary; skriba.eszter@edu.pte.hu (E.S.); tandor.zoltan@edu.pte.hu (Z.T.); szmatona.gabor@edu.pte.hu (G.S.); 5 Department of Obstetrics and Gynaecology, Medical School, University of Pécs, 7624 Pécs, Hungary

**Keywords:** reproductive knowledge, health literacy, reproductive age, women, hormones, ovulation, menstrual cycle, pregnancy, birth control, health inequalities

## Abstract

Background/Objectives: To support women’s informed decisions and reproductive self-care, confident reproductive health-related knowledge is needed, supported by adequate health literacy (HL). No corresponding survey has been carried out in Hungary on inequalities to provide information addressing education. Materials and Methods: In the current cross-sectional online survey, 301 women of reproductive age (27.16 ± 0.36 years) were asked with the Hungarian versions of validated and standardised questionnaires about reproductive knowledge on hormones, ovulation, menstrual cycle, pregnancy signs and birth control (Knowledge of Female Body Scale—KFB), and HL (Brief Health Literacy Screening Tool—BRIEF). Spearman correlation and multivariable linear regression analyses were utilised, with a significance level set at *p* < 0.05. IBM SPSS version 28.0 (IBM SPSS, Armonk, NY, USA: IBM Corp.) and G*Power (version 3.1.9.7; Heinrich-Heine-Universität Düsseldorf, Düsseldorf, Germany) software. The STROBE checklist was followed. The Clinical Trial Registry Nr. is NCT06146673. Results: The KFB composite score was high (20.01 ± 2.33); 86.374% had “high knowledge”. Still, lacking information was identified for the mechanisms of certain contraceptive methods and early physical signs of pregnancy. A significant difference was also found in the KFB scores in the case of higher age (*p* = 0.019), higher education level (*p* = 0.018) and previous live birth (*p* = 0.028). A positive correlation was found between KFB and HL (*p* < 0.001), education (*p* = 0.005), and age (*p* = 0.021). A multiple regression analysis (R^2^ = 0.087, *p* < 0.001) indicated that both HL (*p* < 0.001) and age (*p* = 0.003) are potential positive predictors of adequate reproductive knowledge, whereas induced abortion (*p* = 0.013) might serve as an inverse predictor. Conclusions: Inequalities in women’s knowledge about their reproductive system and HL were found, and it was significantly the lowest in their highest conception probability age. Therefore, in addition to targeted education, HL also needs improvement.

## 1. Introduction

The shift in demographic dynamics is characterised by a robust decline in total fertility rates, so the mean rate of children per woman during their reproductive age in certain populations constantly decreased for decades [1], especially in Europe [2]. Although the global fertility rate stands at 2.3 live births per woman, more than 50% of the countries are not able to maintain a constant population size in the long run. The process is more prevalent in high- and middle-income countries, showing total fertility rates (TFR) below the population replacement level [3,4,5]. In 2022, the TFR was 1.46 among the EU member states and 1.56 in Hungary [6,7,8,9].

More advanced maternal age is one of the confounders of decreasing TFR. The spread of contraceptive pills, female employment, university education, late leaving the parental home, delayed partnership formation, and marriage are considered to be concurrent with the rise of the mean age of primiparas [10]. Due to societal and lifestyle changes and competing priorities [11], it reached 28–29 years in most European countries. In Central Eastern Europe, the mean age of primipara was considerably younger [10], but with a steady increase, it almost reached the European Union average (29.7 years). For example, in Hungary, it still continues to increase slowly, passing the age of 29.24 in 2023 [12].

The delayed family formation has not only a demographic but also a medical perspective as well [10]. Postponed childbirth is highly accompanied by an increased rate of involuntary childlessness and smaller families, since women’s advancing age exposes important non-modifiable medical risk factors [10,13,14,15,16,17]. Age-related decline in female fecundity and a higher rate of adverse events may manifest in ovarian ageing, a longer time to pregnancy, a higher prevalence of infertility, spontaneous abortions, ectopic pregnancy, congenital malformations and trisomy 21, preterm births, stillbirths, or more complications or interventions at birth [10,13,16,18,19,20,21].

The reproductive age of women was defined by the World Health Organization as 15–49 years [22], but the chances to conceive and the occurrence of adverse outcomes are not consistent. There is an 85%, 75%, 66%, and 44% chance of conceiving within one year under 30, at 35, 40, and 45 years, respectively [23]. The delay in attempts of pregnancy starts at around 30 years and becomes clinically relevant by the mid-30s, concerning longer time to pregnancy, infertility, missed abortions and ectopic pregnancies. The risk of preterm or stillbirth increases after 35 years and is more pronounced in the 40s [10]. Even medically assisted reproduction (MAR) cannot completely counter the ageing process in fecundity; the success of the treatments also decreases exponentially with advancing age [10,17]. Male advanced age may also contribute to the deterioration of reproductive capacity, in addition to females, but it has a less pronounced effect on infertility and adverse outcomes [10,16]. Although having a child should be a joint decision in a couple’s life, women’s knowledge is essential in light of the above.

Knowledge about factors that influence fertility is very important to decrease reproductive health concerns of women of reproductive age, to support informed decisions in family planning, and to boost reproductive self-care. School education regarding reproductive health traditionally focuses on avoiding unintended pregnancies and sexually transmitted diseases [24]. Some fertility knowledge surveys proved that there is still a gap. A significant proportion of affected respondents underestimate the age at which fertility starts to decline, have poor knowledge of the biological background, the regulatory role of hormones, and lifestyle factors, and have an inadequate understanding of the menstrual cycle, ovulation, and the likelihood of conceiving or different opportunities in contraception and fertility treatments [23,25,26,27,28,29,30,31,32].

Making informed health decisions is important not only regarding reproduction but also in general and for disease-specific health issues as well [33,34,35,36,37]. Obtaining, processing, and understanding health information and services is consequential to our health decisions; a suitable level of health literacy is necessary [38,39]. Health literacy may play a crucial role in reproductive knowledge, affecting behaviours and outcomes as well. Contraception, fertility, prenatal screening, and sexually transmitted infections (STIs) were proven to have a relationship with health literacy [40].

Limited health literacy is associated with decreased knowledge of the meaning, mechanism of action, and risks of oral contraception [41], difficulties in the practical application of contraceptives [42], choosing less effective methods [43], poorer understanding of the rationality of emergency contraception [44], or on the other hand, with indistinct knowledge about the timing of fertility [43]. In contrast, a high health literacy level is associated with more excellent knowledge about the effect of ageing on fertility and the use of MAR technology [45].

Health literacy was measured in fertility knowledge studies, mainly with the Rapid Estimate of Adult Literacy in Medicine (REALM) [46], reporting patients’ literacy skills, or with the Short Test of Functional Health Literacy in Adults (S-TOFHLA) [47] for reading medical texts and interpreting numeracy sections. With the Newest Vital Sign (NVS) [48] the researcher addresses a task, for patients to interpret a nutrition label, and with the Set of Brief Screening Questions (SBSQ) [49], the confidence in reading hospital materials, filling out medical forms, and learning about medical conditions can be measured. There were already efforts to examine health literacy in Hungary using the previous tools but on the general population [48,49,50].

Many recent societal, lifestyle, and environmental trends have led to the need for fertility education, including the age at which individuals become parents, the development of new reproductive technologies, and family diversity [51]. However, education needs to be based on the current health literacy and reproductive health knowledge level of the affected population. No corresponding survey has been carried out in Hungary so far. To fill these gaps, we aimed to present a cross-sectional study investigating the association between women’s health literacy and knowledge about their reproductive system in reproductive age. The objectives of this study were to describe the level of reproductive knowledge and health literacy and to identify the areas of lacking knowledge for targeted educational purposes, to examine the socio-economic factors associated with inequalities in reproductive knowledge and health literacy, and to reveal the contribution of health literacy to reproductive health knowledge.

## 2. Materials and Methods

### 2.1. Study Design, Settings, and Duration

In September and October 2023, via the Google Forms application, an online cross-sectional survey was conducted using convenience sampling for participant selection. An open-access, self-administered questionnaire was distributed to potential participants through social media (Facebook and Instagram) in women’s health groups. The invitation included the online link to the survey, a detailed information sheet in plain language on the aims of the study, investigators, contact information, and the consent form.

### 2.2. Sample Size, Sampling Method, and Inclusion and Exclusion Criteria

The inclusion criteria for the study specified that participants had to be Hungarian-speaking adult women of reproductive age. Additionally, only those who voluntarily provided their informed consent were included in the study. In alignment with cultural, legal, and biomedical considerations, reproductive age in this study was defined as starting at the age of majority (18 years) and extending up to 45 years. This range is supported by Leridon’s model, which indicates that, by age 45, 78.9% of women attempting pregnancy will remain childless [52]. Current pregnancy, lactation, or contraceptive use were not considered as exclusion criteria. Any participants with cognitive impairments that could interfere with their ability to understand and complete the survey, who had previously participated in similar research or interventions related to reproductive health, or who had substantial missing data may have been excluded.

Through social media platforms, the call for participation and the questionnaire were distributed to circa one thousand five hundred potential participants. A total of three hundred and one respondents gave consent and filled out the questionnaire; the data of which had been fully processed. There was no need for exclusion.

### 2.3. Data Collection Tools, Validation, and Study Variables

The survey included questions to measure social-demographic and educational, anthropometric, general health, and women’s health-related questions. The main objectives were measured with questions on sexual, contraceptive, and health-promoting behaviours, pregnancy-related aspects, and women’s knowledge of their reproductive system.

Through anthropometric questions, similar to the Women’s Health Initiative [53], respondents were asked to report their current weight in kg (“What is your current weight in kg?”), and instructions were given to define their weight to the nearest 0.1 kg using a digital scale without shoes in light clots. Height was asked to be reported (What is your height in cm?) following the given instructions, measured to the nearest 0.1 cm, without shoes, and using a wall-mounted altimeter. Participants’ body mass indexes (BMI) were calculated using the formula provided by the World Health Organization (WHO) [54].

The Knowledge of the Female Body (KFB) Scale, designed by Ayoola et al. [55] was used to assess their knowledge about the female reproductive system, including the reproductive organs, importance of sex hormones, length of menstrual cycle and associated physical symptoms, meaning and timing of ovulation, recognition of early signs of pregnancy, and mechanism of action of barrier and hormonal birth control methods. The KFB consists of 17 questions encompassing 26 possible responses. “Yes”, “No”, or “Don’t know” responses are available. The composite KFB score was categorised as low knowledge, in case of <70% correct responses (0–17 points); 70% or more correct responses (18–26 points) were considered as high knowledge [55].

The Hungarian version of the KFB was adapted to the Hungarian language using the guidelines of Beaton et al. [56]. Two experts in medical English developed the language adaptation of the questionnaire, and then, two independent translators prepared the back translation. After the translation and back translation, inaccuracies and discrepancies were harmonised. A pre-test of the questionnaire involving 30 Hungarian female university students (age 22.34 ± 2.31 years) revealed no interpretation difficulties; it was considered suitable for further testing. Psychometric properties were measured with the involvement of 291 women of reproductive age, which proved the validity and reliability of the questionnaire without any changes in the questionnaire. The current version of the KFB used had a Cronbach alpha coefficient of 0.88. The publication of the validation study is in progress.

The validated Hungarian version of the Brief Health Literacy Screening Tool (BRIEF) was utilised for measuring health literacy (HL) levels. The tool was tested in the general population in a nationwide cross-sectional study, and the internal consistency scored 0.87, measured by the Cronbach-α [57]. The free-to-use scale was created in 2006 and adapted into Hungarian in 2021 [58,59]. It is self-administered and measures health literacy subjectively through four questions. Three of the four items were based on the Chew questionnaire [42] and asked about difficulties in understanding official health documentation and health flyers and filling out forms on their own health status. One more question concerns the comprehension of verbal information. It is calculated by summing up the answers given on a five-point Likert scale (1–5) to determine the total score (4–20 points); the respondents’ summed answers can be categorised as inadequate (4–12), marginal (13–16), or adequate (17–20) health literacy [57,58].

### 2.4. Statistical Analyses

The reporting method used in this study adhered to the Strengthening the Reporting of Observational Studies in Epidemiology (STROBE) checklist [60]. The descriptive statistics were calculated and presented as the mean and standard deviation (SD) or frequency (%). The Kolmogorov–Smirnov test was used to evaluate the distribution of the data. Mann–Whitney U tests, Kruskal–Wallis tests, and Spearman’s rank correlations were used to test the predictors of the KFB scores using bivariate analysis. Multivariate linear regression was implemented to test the effect of demographic parameters, health indicators, and health literacy on the KFB scores. The significance level for all tests was set at *p* < 0.05. Statistical analyses were performed using IBM SPSS Statistics for macOS, version 28.0 (IBM SPSS, Armonk, NY, USA: IBM Corp.). The sample size required, based on the multivariate linear regression model, was calculated by estimating an effect size of 0.20 and adopting a significance level of 0.05, with a statistical power of 0.80. Therefore, we aim to recruit, at minimum, a sample of 91 participants. G*Power software (version 3.1.9.7; Heinrich-Heine-Universität Düsseldorf, Düsseldorf, Germany) was used for sample size calculation.

### 2.5. Ethical Considerations

Before the query, the survey was approved by the Medical Research Council of Hungary (Identifier: BM/23899-1/2023) and registered on the Clinical Trial Registry (NCT06146673registration date date. 23 November 2023). The study was conducted in line with the Declaration of Helsinki [61]. To maintain the respondents’ anonymity, no identifying information (names, usernames, email addresses, token fields, etc.) was captured in the survey responses.

## 3. Results

### 3.1. Descriptive Data

To some extent, the reported sample showed a higher socio-demographic status than the Hungarian average. Compared to the relatively young age (27.28 ± 6.09 years) of the respondents, a high level of higher education was characteristic in the sample; 55.2% had a college or university degree. However, only one-third (30.23%) were single. Others lived with a partner in marriage or cohabitation, and only 18.9% lived in rural areas.

From an economic point of view, their status was considered relatively modest. More than half of them (53.49%) self-rated their financial status as below average, where the average was considered to be 1440 EUR according to the Hungarian Central Statistical Office [62]. This may be related to the distribution of the sample, since 119 still-studying women were questioned, which may also explain the high non-employed rate (34.55%). Most respondents who were active in the labour market were occupied as professional workers or administrative employees (47.17%).

Not only was their self-reported health status positive, in toto, 86.37% considered their SRH about the same or better compared to their age peers, where 68.44% had a healthy weight, with a BMI of 18.5 to less than 25 kg/m^2^. Although more than 83.72% reported occasional or weekly alcohol and daily caffeine consumption, 2.99% tried drugs, and 71.76% took prescribed medication or dietary supplements (See Table 1).

Women of reproductive age had 86.37% high knowledge of their reproductive system based on the KFB composite scale and 83.38% adequate or marginal health literacy based on the Brief Health Literacy Screening Tool (Table 2).

In general, the knowledge of the study population about their reproductive system was high; 79.54% of all answers were true on the KFB Scale. Only 8.06% of the questions were answered “I don’t know”. The percentage of respondents belonging to the “high knowledge” group was 86.37%, having at least 70.00% correct choices, and they reached 20.01 ± 2.33 on the composite KFB score.

Nevertheless, there were critical topics for which a high proportion of incorrect answers were received. For “Headache could be an early sign of pregnancy”, 84.33% of the respondents answered incorrectly. Seventy per cent thought that the vaginal ring is not a barrier method, and according to 59.90% of them, it prevents the ovum from maturing and being released. They also had difficulties (57.33%) with the statement that “Some women with irregular periods may become pregnant at any time of the menstrual cycle”. Among contraceptive methods, they were not confident with the mechanisms of injections/shots (60.33% correct answers), diaphragm (66.33% correct answers), and even birth control pills (68.67% correct answers). Only 63.67% had proper knowledge that the light period could be an early sign of pregnancy, and 68.00% knew that “an egg lives for only 1 day, but a man’s sperm lives in a woman’s body for 5–7 days”.

However, they had confident knowledge of the importance of missed periods as early physical signs (100.00%) and that the uterus (100.00%) and ovary (99.33%) are needed structures in a woman’s body to be able to have babies. Most of them (99.33%) also knew that checking on ovulation can help to both plan and prevent pregnancy, and in the same context, “If a woman has sex during ovulation, she could get pregnant” (98.67%). They have the best knowledge of the contraceptive methods of condoms (99.00%).

Regarding health literacy, they scored 15.46 ± 3.00 based on the Brief Health Literacy Screening Tool. Understanding flyers prepared for laypeople and oral information about their health status caused no difficulties for the respondents in general. Filling out medical forms made them less confident, and they needed the most support to understand written medical documents (e.g., outpatient cards and final reports).

Dividing the results by age groups (*p* = 0.019) and education level (*p* = 0.018), higher female-body knowledge and health literacy scores were found with older age and higher educational levels, but it reached the statistical significance by KFB only (Table 3).

### 3.2. Bivariate Analysis

Investigating the differences between the groups with or without existing health or risk behaviours and reproductive health issues, the Mann–Whitney U Test revealed significant mean differences regarding previous live births with the knowledge of the female reproductive system. Knowledge was higher in the case of parity (19.90 ± 2.28 vs. 20.75 ± 2.50, *p* = 0.028), and in the case of taking prescribed medication regularly, the health literacy level was higher (14.94 ± 3.02 vs. 15.66 ± 2.97, *p* = 0.046) (Table 4).

The relationship between knowledge of the reproductive system, health literacy, and certain socio-demographic, economic, and SRH parameters was tested with a bivariate analysis. A positive relationship was found between reproductive knowledge and health literacy (*p* < 0.001) and the highest level of education (*p* = 0.005) and age (*p* = 0.021). The health literacy level increased along with the size of the settlement (*p* = 0.033). All correlations remain weak (Table 5).

### 3.3. Multivariate Analysis

A multiple linear regression analysis was conducted to predict the influencing factors of the knowledge of the reproductive system (composite KFB score). As shown in Table 6, the multiple linear regression model (R2 = 0.087, *p* < 0.001) showed that reproductive knowledge was significantly predicted by health literacy (one unit of the HL scale means a 0.265 increase on the KFB scale, *p* < 0.001), age (one year increase of age means 0.098 points more on the KFB scale, *p* = 0.003), and induced abortion (a previous abortion means a 2.387 point decrease in the KFS score, *p* = 0.013).

## 4. Discussion

To support women’s informed decisions and reproductive self-care, confident reproductive health-related knowledge is needed. On this behalf, women of reproductive age need to have adequate knowledge about the female reproductive system. Proper health literacy may also help to allay the reproductive health concerns of women by obtaining, processing, and understanding fertility-related health information effortlessly. Since the knowledge of the studied population is still insufficient, it could affect reproductive behaviours and outcomes [40]. Up-to-date and evidence-based fertility education sources may improve reproductive knowledge and decisions and decrease infertility incidence and reproductive treatment needs [51]. This addressed education needs to be provided at the current health literacy level to be internalised more easily. To investigate the level of knowledge regarding the female body (basic anatomy, physiology, and the possibilities of conceiving or contraception) and its influencing factors with special regard to health literacy, a cross-sectional study was conducted among Hungarian women of reproductive age.

In the current study population, the knowledge was seen as confident in most areas. In general, the knowledge of the study population about their reproductive system was high. Belonging to the “high knowledge” group were 86.4% of respondents. Still, some focal points that lacked information were identified. A significant difference was found in the Knowledge of the Female Body Scale scores in cases of higher age, higher education level and previous live birth. A significant positive correlation was found between KFB and the following factors: health literacy, education, and age. In the final multiple regression model, it was also revealed that, even though health literacy and age, as primary and secondary influencing factors, are positive, induced abortion has an inverse relationship, and it implies limited reproductive knowledge. Health literacy was higher in the case of taking medications regularly and having an urban residence.

Different results were found in our sample compared to the study of Ayoola et al., who investigated women of the same age but with a different culture (mostly Hispanic and African American) and a more modest socio-economic situation, using the same tool, namely the Knowledge of the Female Body Scale. In this study, women were more characterised by “low knowledge” (68.00%) of female reproduction and its associated changes, compared to our study (13.62%). The average composite KFB score was lower (15.10 ± 5.09), and age had a relevant but not significant inverse relationship with reproductive knowledge in these low-income US women. If we consider the ratio of proper answers measured by the KFB, some parallel results were still found in the US and Hungarian studies on the basic anatomy of the female body. A high percentage of respondents knew that ovaries (86.4% vs. 99.33%), fallopian tubes (78.40% vs. 98.00%), and the uterus (82.40% vs. 100.00% respectively) are needed structures to conceive and give birth. Both samples were informed about the importance of missing periods as an early physical sign of pregnancy (90.40% vs. 100.00%), and they were both aware that, if women have sex during ovulation, they could get pregnant (75.20% vs. 98.67%). To a certain extent, incompetent answers were reported concerning reproductive physiology regarding the lifespan in the body (37.60% vs. 68.00%) and the number (20.80% vs. 73.67%) of eggs and sperm. Both samples had difficulties with identifying a light period as an early pregnancy sign and realising that women with irregular periods may become pregnant at an indeterminable time during the menstrual cycle (45.60% vs. 42.67%). This last topic proved to be the Achilles heel of the Hungarian respondents. They achieved the worst result in relation to this question, and their knowledge in this single case was lower than that of the US sample.

However, concerning the definition (91.00% vs. 52.80%) and time of ovulation (90.67% vs. 32.80) or the function of hormones, as to their role in the pregnancy (96.00% vs. 53.60%) and menstrual cycle (96.67% vs. 30.40%) the European sample scored quite higher compared to the American one. The last value relates to the proper answers related to the oestrogen hormone, but their knowledge was even lower in relation to the progesterone hormone, with 24.00% proper answers, which indicates the largest knowledge gap in this population.

Health literacy was not measured by the above sample, and the authors did not report the exact educational level of the respondents, only that they came from a notably low-income community, which could have a connection to insufficient education and cause the significant difference in the level of knowledge and which topics indicated lack of knowledge in the samples. Gazmararian et al. demonstrated in a low-income population that a lower level of education and, due to this, lower reading skills and family planning knowledge and practices are correlated. They also investigated women of reproductive age (N = 406) and found, using logistic regression adjusted for age, race, and marital status, that women with low reading skills were 2.2 times (95% CI 1.1, 4.4) more likely to seek information on contraceptive methods and 4.4 times (95% CI 2.2, 9.0) more disposed to have insufficient knowledge about when they were most likely to get pregnant [43].

The importance of education was underlined in several studies [26,28,63,64,65,66]. In line with a higher level of education, as was characteristic in our sample, a higher knowledge of the biological aspects of reproduction (sex hormones, definition of ovulation) may be related to the fact that they acquired more information during regular education. In relation to school education, sex education should also be discussed. The proportion of correct answers suggests, in line with several studies, that school sex education focuses primarily on avoiding STDs and unwanted pregnancy. In line with this, in our study, the highest knowledge about contraceptive methods was given in the case of condoms, where 99.0% identified it as a barrier method. At the same time, 93% recognised by the counter test that it does not prevent the maturation and release of the egg. However, only 30.0% realised that the vaginal ring was not a barrier method, and only 40.1% knew that it was a type of hormonal contraception.

Despite the combined socio-economic status and residency, Hammarberg et al. reported low-to-average reproductive knowledge based on the self-estimation of the participants in a qualitative study [25]. These couples of reproductive age, who were contemplating or preparing for pregnancy, were asked about the optimal time of intercourse, and this question revealed that their knowledge regarding women’s menstrual cycle and ovulation was generally low. They were aware that there is a “window of time” for a woman to conceive. However, details regarding when this occurs were deficient. However, in our study, participants gave more than ninety per cent proper answers on the definition and time of ovulation. All they had to do was only to choose whether a given statement was correct or incorrect. It is conceivable that relying solely on their own prior knowledge, they would be more uncertain and would not give such a large proportion of correct answers in their own words. Difficulties in the identification of ovulation [26,67,68] or moderate awareness of the fertile period [65,69,70] were observed by previous studies in the general population in diverse cultural contexts. Based on a current review of fertility awareness [29], a higher awareness level was found only in the case of pre-existing involvement, such as infertility [71,72], or professional knowledge, as in the case of nurses [73].

In our study, health literacy seemed to have a dominant contribution to higher reproductive knowledge. It is also verified by a systematic review by Kilfoyle et al. [40], which states that HL is related to reproductive knowledge across a spectrum of topics, as a higher level of HL was associated with more adequate knowledge of the proper use of oral contraception [41] or emergency contraception [44], the time when they were able to conceive during the cycle [74], and age-related fertility decline and the need for MAR [75,76].

Nevertheless, our study outlined that age is the second most important factor regarding reproductive knowledge. The knowledge is significantly higher in advanced age, which suggests that women used to seek information on their own or learn based on their experience. Similar conclusions can be drawn based on a Swedish randomised controlled intervention trial on a higher level of knowledge based on four basic questions on the lifespan of an ovum, chance of pregnancy by a specific age, fertility decline by age, and success rate of MAR [64]. Other studies also supported the relationship between better knowledge or awareness and advanced age [28,45,64,70].

The knowledge was also significantly higher in the case of previous live birth (*p* = 0.028) in our study. Pedro et al. reported controversial results in a systematic review, which reported significantly more studies with no effect regarding childbearing status based on five studies versus two studies, proving that parents have better reproductive knowledge [29].

However, induced abortion proved to have a negative relationship with knowledge. From the above, we can conclude that proper counselling is still not provided for younger generations. This situation suggests that the already widely, even online available, educational opportunities are not sufficient to boost reproductive knowledge and design a reproductive life plan, and although this awareness of the effect of ageing on reproductive potential was not measured in the present research, they may run out of time when making decisions or when trying to fulfil them.

Women’s knowledge about their reproductive system was found to be significantly (*p* = 0.019) the lowest (19.84 ± 2.05) in their highest conception probability period, namely in the 18–25 years age group in the current study. HL, as a determinant of women’s reproductive knowledge, was also the lowest in the same age group. Therefore, in addition to the sharing of targeted information that develops the reproductive knowledge of this group, more general education aimed at improving health literacy also needs development and could support the primary goal, namely the improvement of reproductive knowledge [75].

Inadequate preventive programs and insufficient health literacy can lead to an increased prevalence of sexually transmitted infections (STIs), which have significant consequences for reproductive health [76]. Some of these STIs have harmful effects on reproductive health, such as Chlamydia trachomatis and Neisseria gonorrhoeae. Which may cause pelvic inflammatory disease (PID) in women, leading to damage of the fallopian tubes, infertility, and an increased risk of ectopic pregnancy [77] or damage to the cells responsible for transporting fertilised eggs, thereby reducing the likelihood of successful conception [77,78]. Due to increasing antibiotic resistance, management is even more challenging, and to avoid reproductive health risks, prevention would be even more important.

In Hungary, the National Public Health Program 2023–2033 [79] only discusses this issue in terms of the high-risk sexual behaviours of high school students, mentioning that 43.00% of them are sexually active and 20.00% of them did not protect themselves in any way during the last sexual encounter, which is a relatively high risk in international comparison. The document also mentions the prevalence of Treponema Pallidum, Neisseria Gonorrhoea, human immunodeficiency virus, hepatitis B virus, and human papillomavirus infections in Hungary and sets the target values for prevalence and (where this can be interpreted) vaccination rates in accordance with the WHO recommendations and the recommended interventions, including the necessary personal, material, professional, and organizational interventions. Despite this, campaigns aimed at stimulating reproductive awareness for the prevention of sexual and reproductive health or a comprehensive reproductive health program are not well known. Our research group just applied to contribute to the International Reproductive Health Education Collaborations (IRHEC) call [51,80,81] for the development and dissemination of free educational resources aimed at reducing the incidence of infertility and promoting the health of future generations by enhancing reproductive health education (www.eshre.eu/IRHEC, date of access: 10 August 2024). These resources may be a part of future implications for improving reproductive awareness in Hungary.

In summary, the study’s strengths include that it was the first comprehensive study in Hungary to assess reproductive knowledge in a detailed manner regarding female reproductive anatomy, the function of sex hormones, and the specificities of the menstrual cycle, ovulation, and conception, enabling the identification of gaps in knowledge and understanding among Hungarian women. Another strength may be that it focused on reproductive knowledge across different age groups, which allowed the study to highlight differences in understanding based on age and reproductive experiences, offering valuable insights for future health interventions.

Despite the above, it presents the following limitations. The moderate sample size, the online recruitment of participants, and the convenient sampling method warn against generalising the results. Our findings cannot be extrapolated to all women of reproductive age, taking into consideration the heterogeneity in their educational background (field of study), and due to the underrepresentation of women with low educational and socio-economic levels. Our results could also be biased by the fact that the respondents were invited through women’s health groups, and therefore, they may be particularly interested in their reproductive health and have a higher level of knowledge. Due to the gender selection of the study, any estimation of men’s knowledge can be drawn. Lacking information on the number of children, wanted or unwanted pregnancies, current child wish, religious beliefs, and other factors could have influenced our results, which implies a further, more detailed investigation and special intention to measure the awareness of age-related fertility decline.

## 5. Conclusions

Women’s knowledge about their reproductive system was found to be relatively high among Hungarian women of reproductive age but, still, lacking information could be defined for certain reproductive health topics. The study’s comprehensive methodology ensured that it captured various facets of reproductive health, enabling the identification of gaps in knowledge and understanding among Hungarian women. This could lead to targeted educational and public health initiatives to improve reproductive health awareness in the population specified by age and reproductive experience. The results could serve as a good basis to build up extended sexual education for adults, counteract the consequences of too-early or late family formation, and support informed decisions and reproductive self-care, especially in the highest conception probability age with suggested programs.

In addition to targeted education on lacking reproductive information, HL also needs to be improved. With the above-mentioned measures, socio-economic and health literacy inequalities, as determinants of women’s knowledge about their reproductive system, may become more balanced.

## Figures and Tables

**Table 1:** Socio-demographic, anthropometric and health characteristics of study participants (N = 301).

Socio-Demographic Characteristics			
		N	%
**Highest level** **of education**	Primary education	1	0.33%
	Vocational training	2	0.66%
	Secondary school	17	5.65%
	High school	115	38.21%
	BSc/BA (college degree)	93	30.90%
	MSc/MA (university degree)	73	24.25%
**Marital status**	Married, lives with spouse	88	29.24%
	Married, non-cohabitating	1	0.33%
	Cohabitation relationship	121	40.20%
	Single	91	30.23%
**Type of residence**	Capital city	72	23.92%
	County seat, city	114	37.87%
	Town	58	19.27%
	Village, farm	57	18.94%
**Type of occupation**	Non employed	104	34.55%
	Auxiliary worker, trained worker	18	5.98%
	Skilled worker	10	3.32%
	Professional worker	93	30.90%
	Administrative employee	49	16.28%
	Managerial position	27	8.97%
**Self-rated** **financial status**	Below average	161	53.49%
	Average	86	28.57%
	Above average	54	17.94%
**Anthropometric and Health Characteristics**			
		Mean	SD
**Anthropometrics**	Age (years)	27.28	6.09
	Height (cm)	167.13	6.56
	Weight (kg)	63.58	11.50
	BMI (kg/m^2^)	22.76	3.92
		N	%
**BMI Categories**	Underweight. Less than 18.5	25	8.31%
	Healthy Weight. 18.5 to less than 25	206	68.44%
	Overweight. 25 to less than 30	52	17.28%
	Obesity. 30 or greater	15	4.98%
	Class 1 Obesity. 30 to less than 35	3	1.00%
**Self-rated health** **compared to age peers**	Much worse	4	1.33%
	Worse	37	12.29%
	About the same	149	49.50%
	Better	90	29.90%
	Much better	21	6.98%
**Health/risk behaviour**	Smoking	56	18.60%
	Alcohol consumption	252	83.72%
	Caffeine consumption	251	83.39%
	Use of drugs	9	2.99%.
	Use of medication	216	71.76%
**Reproductive health** **issues**	Current pregnancy	10	3.32%
	Current lactation	7	2.33%
	Previous live birth	38	12.62%
	Induced abortion	14	4.65%
	Irregular periods	67	22.26%
	Intermenstrual bleeding	37	12.29%
	Menstrual cramps	257	85.38%
	Diagnosed PCOS	49	16.28%
	Use of hormonal contraception	61	20.27%

Abbreviations: BMI = body mass index; PCOS = polycystic ovary syndrome; SD = standard deviation.

**Table 2:** The knowledge of women of reproductive age about their reproductive system and health literacy by knowledge and literacy categories based on Knowledge of the Female Body Scale and the Brief Health Literacy Screening Tool (N = 301).

Scale	Category	N	%
**Knowledge of the** **Female Body Scale**	Low knowledge	41	13.62%
	High knowledge	260	86.37%
**Brief Health Literacy** **Screening Tool**	Inadequate	50	16.61%
	Marginal	128	42.52%
	Adequate	123	40.86%

**Table 3:** The knowledge of women of reproductive age about their reproductive system and health literacy by age and education categories based on Knowledge of the Female Body Scale and the Brief Health Literacy Screening Tool (N = 301).

	Knowledge of the Female Body Scale	Brief Health Literacy Screening Tool
Total (N = 301)		
Mean (SD)	20.01 ± 2.33	15.46 ± 3.00
Range	(11–25)	(6–20)
**Age**		
18–25 years (*n* = 152)	19.84 ± 2.05	15.37 ± 2.92
26–35 years (*n* = 119)	19.98 ± 2.55	15.50 ± 3.02
36–45 years (*n* = 30)	20.96 ± 2.54	15.77 ± 3.38
*p*	0.019 *	0.681
**Education**		
Primary (*n* = 1)	19.93	10.00
Secondary (*n* = 134)	19.57 ± 2.33	15.29 ± 3.02
Higher (*n* = 166)	20.37 ± 2.78	15.63 ± 2.96
*p*	0.018 *	0.172

Abbreviations: SD = standard deviation. Kruskal–Wallis H test, * *p* < 0.05.

**Table 4:** The knowledge of women of reproductive age about their reproductive system and health literacy between-group differences by health and risk behaviours and reproductive health issues based on Knowledge of the Female Body Scale and the Brief Health Literacy Screening Tool (N = 301).

		Knowledge of the Female Body		Health Literacy	
		Z	*p*	Z	*p*
**Health/risk** **behaviour**	Smoking	−0.881	0.378	−1.026	0.305
	Alcohol consumption	−0.750	0.453	−0.679	0.497
	Caffeine consumption	−0.185	0.853	−0.402	0.688
	Use of drugs	−0.935	0.350	−0.309	0.757
	Use of medication	−1.525	0.127	−2.000	0.046 *
**Reproductive** **health** **issues**	Current pregnancy	−1.189	0.234	−0.898	0.369
	Current lactation	−0.492	0.623	−1.229	0.219
	Previous live birth	−2.195	0.028 *	−0.022	0.982
	Induced abortion	−0.980	0.327	−1.398	0.162
	Irregular periods	−0.245	0.807	−1.289	0.197
	Intermenstrual bleeding	−0.366	0.715	−1.644	0.100
	Menstrual cramps	−0.827	0.408	−1.934	0.053
	Diagnosed PCOS	−0.363	0.717	−0.269	0.788
	Use of hormonal contraception	−1.032	0.302	−0.062	0.950

Abbreviation: PCOS = polycystic ovary syndrome. Mann–Whitney U test, * *p* < 0.05.

**Table 5:** The relationship between knowledge of the reproductive system, health literacy, and certain socio-demographic, economic, and SRH parameters among women of reproductive age (N = 301).

	Knowledge of the Female Body		Health Literacy	
	R	*p*	R	*p*
**Knowledge of the female body**			**0.222**	<0.001 **
**Health literacy**	**0.222**	<0.001 **		
**Highest level of education**	**0.163**	0.005 **	0.071	0.219
**Marital status**	0.001	0.985	0.002	0.979
**Type of residence**	−0.109	0.059	**−0.123**	0.033 *
**Type of occupation**	0.090	0.119	0.009	0.878
**Self-rated financial status**	0.076	0.191	0.076	0.189
**Age (years)**	**0.133**	0.021 *	0.057	0.327
**BMI**	0.074	0.203	−0.048	0.408
**Self-rated health compared to age peers**	0.098	0.088	0.036	0.539

Abbreviation: BMI = body mass index. Spearman’s rho, * *p* < 0.05, ** *p* < 0.01.

**Table 6:** Multiple linear regression analysis to predict the influence factors of the knowledge of the female reproductive system with stepwise method (N = 301).

Influence Factors	β	95%CI ^a^ Lower	95%CI Upper	Standardised β	T	*p*
**Constant**	23.462	20.827	26.098		17.521	<0.001
**Health literacy**	0.265	0.137	0.393	0.227	4.084	<0.001
**Age**	0.098	0.033	0.163	0.171	2.955	0.003
**Induced abortion**	−2.387	−4.277	−0.498	−0.144	−2.487	0.013

^a^ CI Confidence interval; R2 = 0.087, F = 9.376, *p*  <  0.001. Excluded variables were: marital status, previous live birth, current pregnancy, subjective health status, current lactation, use of medication, and place of living.

## Data Availability

The data presented in this study are available on reasonable request from the corresponding author. The data are not publicly available due to privacy restrictions.
